# Association between antibiotics and dementia risk: A retrospective cohort study

**DOI:** 10.3389/fphar.2022.888333

**Published:** 2022-09-26

**Authors:** Minseo Kim, Sun Jae Park, Seulggie Choi, Jooyoung Chang, Sung Min Kim, Seogsong Jeong, Young Jun Park, Gyeongsil Lee, Joung Sik Son, Joseph C. Ahn, Sang Min Park

**Affiliations:** ^1^ Department of Biomedical Sciences, Seoul National University Hospital, Seoul National University College of Medicine, Seoul, South Korea; ^2^ College of Medicine, Jeonbuk National University, Jeonju, South Korea; ^3^ Department of Internal Medicine, Seoul National University Hospital, Seoul National University College of Medicine, Seoul, South Korea; ^4^ Department of Biomedical Informatics, CHA University School of Medicine, Seongnam, South Korea; ^5^ Medical Research Center, Genomic Medicine Institute, Seoul National University, Seoul, South Korea; ^6^ Department of Family Medicine, Seoul National University Hospital, Seoul National University College of Medicine, Seoul, South Korea; ^7^ Department of Internal Medicine, Hallym University Sacred Heart Hospital, Anyang, South Korea; ^8^ Division of Gastroenterology and Hepatology, Mayo Clinic, Rochester, NY, United States

**Keywords:** dementia, Alzheimer disease, brain‐gut axis (BGA), gut dysbiosis, antibiotics, epidemiology, analytic (risk factors), hazard and risk

## Abstract

**Background:** The possible relation between antibiotic exposure and the alteration of gut microbiota, which may affect dementia risk, has been revealed. However, the association between antibiotics and dementia incidence has rarely been studied. We aimed to determine the association between antibiotic exposure and the risk of dementia.

**Methods:** This population-based retrospective cohort study used data from the National Health Insurance Service-Health Screening Cohort (NHIS-HEALS) in South Korea. Exposure was the cumulative days of antibiotic prescription from 2002 to 2005. Newly diagnosed overall dementia, Alzheimer’s disease (AD), and vascular dementia (VD) were identified based on diagnostic codes and prescriptions for dementia-related drugs. The follow-up investigation was carried out from 1 January 2006 to 31 December 2013. The Cox proportional hazards regression was used to assess the association between cumulative antibiotic prescription days and dementia incidence.

**Results:** A total of 313,161 participants were analyzed in this study. Compared to antibiotic non-users, the participants who used antibiotics for 91 or more days had an increased risk of overall dementia [adjusted hazard ratio (aHR), 1.44; 95% confidence interval (CI), 1.19–1.74], AD (aHR, 1.46; 95% CI, 1.17–1.81), and VD (aHR, 1.38; 95% CI, 0.83–2.30). Those who used five or more antibiotic classes had higher risks of overall dementia (aHR, 1.28; 95% CI, 1.00–1.66) and AD (aHR, 1.34; 95% CI, 1.00–1.78) than antibiotic non-users.

**Conclusion:** Antibiotic exposure may increase the risk of dementia in a cumulative duration-dependent manner among adult participants. Future studies are needed to assess the causality between the long-term prescription of antibiotics and dementia risk.

## Introduction

Dementia is one of the most prevalent neurodegenerative diseases globally (Cao et al., 2020). In 2020, over 50 million people had dementia worldwide, and this number is expected to rise to 152 million by 2050 (Barbarino et al., 2020). Dementia is a leading cause of morbidity, disability, and mortality (Witthaus et al., 1999; Noale et al., 2003; Todd et al., 2013). Alzheimer’s disease (AD) was the sixth greatest cause of death in the United States in 2017 (Kramarow and Tejada-Vera, 2019). Moreover, the total annual global cost is estimated to be the US $2 trillion by 2030 (Barbarino et al., 2020). In this regard, the risk factors such as biological- and lifestyle behavior-related factors are being added by new evidence support consistently (Livingston et al., 2020). Recent studies have indicated that dementia is associated with gut microbiota (Angelucci et al., 2019; Liu et al., 2019; Askarova et al., 2020; Łuc et al., 2021).

Gut microbiota consists of the aggregate of all microorganisms that affect essential human functions (Sekirov et al., 2010; Lange et al., 2016). Specifically, the gut microbiota–brain axis is a bidirectional communication network between the central nervous system (CNS) and the enteric nervous system (ENS) that maintains the homeostasis of the CNS, gastrointestinal, and microbial systems (Carabotti et al., 2015; Kowalski and Mulak, 2019; Morais et al., 2021). In the presence of dysbiosis, the products of pathogenic microorganisms can cross the blood–brain barrier (BBB), and peripheral immune cells activate glial cells and the neuroinflammatory pathway (Doifode et al., 2020). This interplay induces amyloid-beta burden and tau accumulation, triggering neurodegeneration (Sadigh-Eteghad et al., 2015; Doifode et al., 2020). Therefore, the dysbiosis of gut microbiota caused by antibiotics is considered to play an important role to enhance neurodegenerative disease severity (Askarova et al., 2020; Morais et al., 2021).

Previous studies about the relationship between antibiotics and dementia are insufficient. The gut microbiome is highly affected by an unhealthy lifestyle (such as smoking status, irregular physical activity, unhealthy diet, immoderate alcohol consumption, and sleep deprivation) and its significant changes have been reported in patients with AD (Lourida et al., 2019; Askarova et al., 2020). In particular, the changes in the intestinal microbiota were found in elderly patients after antibiotic therapy (Askarova et al., 2020). Moreover, the fecal microbial diversity of AD patients was markedly distinct from individuals with amnestic mild cognitive impairment or healthy controls (Liu et al., 2019). A recent study suggested that gut microbiota changes induced by the use of antibiotics such as streptozotocin and ampicillin can cause AD, whereas broad-spectrum antibiotics including gentamicin and vancomycin may be utilized to treat AD depending on their microbiome target (Zhu et al., 2020). Therefore, a better epidemiological comprehension of the relationship between antibiotics and dementia is needed. In particular, South Korea has a high rate of antibiotic usage, with 31.7 defined daily doses (DDD) per thousand people in 2016, compared to the OECD average of 23.7 (OECD, 2020).

This population-based retrospective cohort study was conducted to assess the association between antibiotic exposure and the risk of dementia incidence using the Korean National Health Insurance Service-Health Screening Cohort (NHIS-HEALS) database.

## Materials and methods

### Study population

The NHIS provides compulsory health insurance for all South Korean, and it covers almost all types of medical services, including health screening for all dependents aged 40 and over biennially. The NHIS-HEALS database is constructed by a 10% random sampling of all individuals aged between 40–79 years who participated in the 2002 and 2003 health examinations, and these participants were followed up until 2013 (Cheol Seong et al., 2017). The database includes basic sociodemographic information, outpatient and inpatient department visits, and pharmaceutical prescriptions (Seong et al., 2017). Diverse epidemiological studies have been conducted using the NHIS database, and its validity has been well proven (Kim et al., 2020).

**FIGURE 1:**
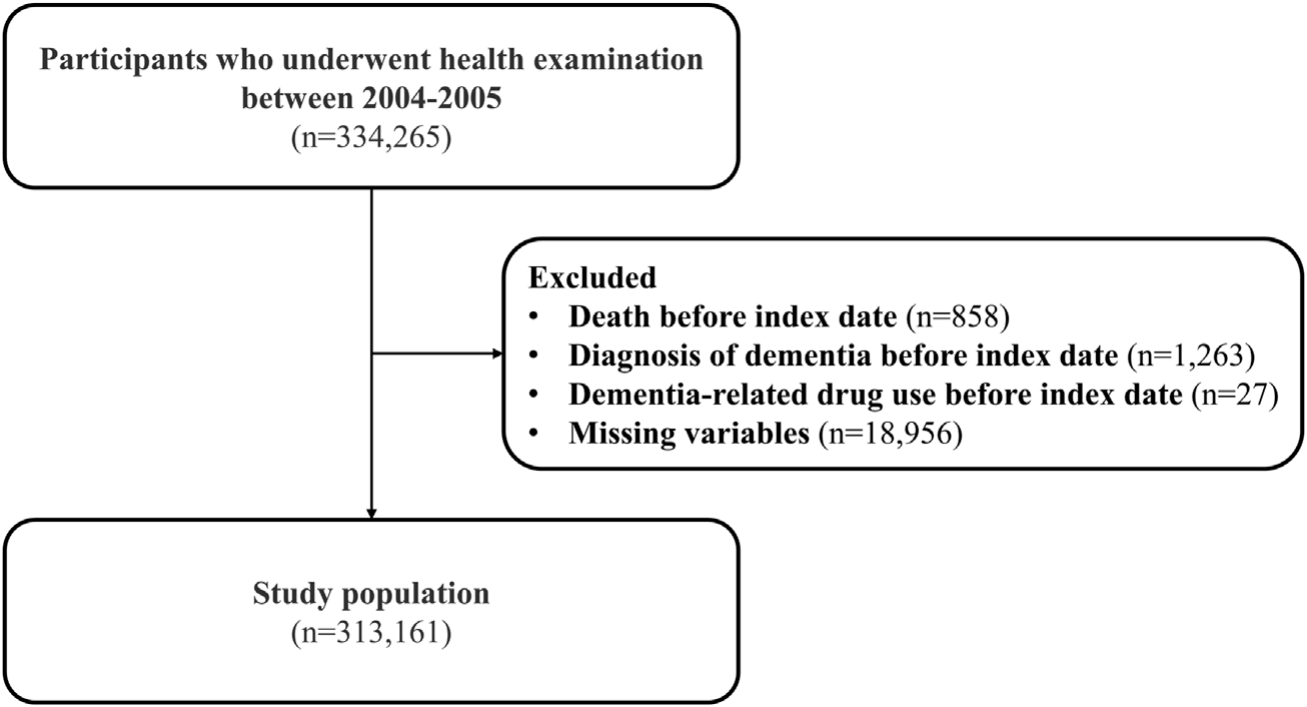
Study sample selection.

Among the 334,265 participants who completed health screening between 2004 and 2005, 858 individuals who died before the index date of 1 January 2006, were excluded. In addition, we excluded 1,290 individuals who had a dementia diagnosis or a prescription of dementia-related drugs before the index date. The 18,956 individuals who had missing covariate values were also excluded (Figure 1). Antibiotic exposure was observed between 2002 and 2005. Starting from 1 January 2006, a total of 313,161 individuals were followed up until they were diagnosed with dementia, died, or 31 December 2013, whichever happened first.

### Key variables

The primary outcome was the development of dementia during the observation period, defined by the 10th Revision of the International Classification of Diseases (ICD-10) codes of F00–F03 and G30 with prescription records of anticholinesterase drugs (memantine, donepezil, rivastigmine, or galantamine).The diagnosis codes for Alzheimer’s disease (AD; F00 and G30) and vascular dementia (VD; F01) were adopted from a previous study (Choi et al., 2018).

The exposure variables were the cumulative days of antibiotic prescription and the number of antibiotic classes between 2002 and 2005, the first 4 years in the database. The cumulative days of antibiotic use were calculated as the sum of all days of antibiotic subscription from 2002 to 2005. Penicillin, cephalosporin, macrolides, fluoroquinolones, sulfonamides, lincosamides, tetracyclines, and vancomycin were defined by the World Health Organization Anatomical Therapeutic Chemical (ATC) classification of drugs. Antibiotic exposure groups were classified as 0, 1–30, 31–90, and 91 or more days of prescriptions. The number of antibiotic classes was classified as 0, 1, 2, 3, 4, and 5 or more.

### Covariates

Covariates were extracted between 2002 and 2005, including age, sex, body mass index (BMI), smoking status, alcohol consumption, physical activity, household income, Charlson comorbidity index (CCI), fasting blood sugar, systolic blood pressure, total cholesterol, infectious diseases (respiratory diseases, urinary tract infections, skin, soft tissue, bone and joint infections, intra-abdominal infections, and other infectious diseases) (Lederberg, 2000; Morens et al., 2004; Morens et al., 2008), and antidepressant use (Kodesh et al., 2019). The household income was classified based on each patient’s health insurance premium. CCI was calculated by the diagnoses for crucial comorbidities (Sundararajan et al., 2004; Quan et al., 2005). Infectious diseases, which are possible comorbidities related to an antibiotic prescription, were included as covariates using the ICD-10 codes (Park et al., 2021). Infectious diseases were adjusted for five sources of infection in the analysis model, not just one variable.

### Statistical analysis

Multivariable Cox proportional hazard regression was performed to estimate the adjusted hazard ratios (aHRs) and 95% confidence intervals (CI) for overall dementia, AD, and VD by antibiotic use. Age, sex, BMI, smoking status, alcohol consumption, physical activity, household income, CCI, fasting blood sugar, systolic blood pressure, total cholesterol, and antidepressant use were considered as potential confounders in the first model. The antibiotic non-user group was considered the reference group in the first model. The second model additionally adjusted for infectious diseases, which are the primary cause of antibiotic usage. In addition, 1–30 days of the exposure group was used as the reference group when evaluating the risk of dementia among antibiotic users only. Stratified analyses of subgroups that were stratified by age, sex, BMI, physical activity, household income, CCI, and antidepressant use were performed. Furthermore, we carried out sensitivity analyses that determined the association of antibiotic use with dementia risk after excluding patients diagnosed with dementia within the first 1–3 years of follow-up. In other words, we did not consider new cases of dementia for those diagnosed within the first 3 years of follow-up to address protopathic bias (Park et al., 2021). We also conducted sensitivity analyses by shifting the index date to 1 year later to minimize possible confounding effects caused by the drug exposure period after the defined index date (Kim et al., 2018). In other words, the analysis was conducted by extending the exposure period from 4 to 5 years. Finally, we conducted a propensity score matching analysis to define the association of antibiotics with dementia risk more precisely. Age, sex, BMI, smoking status, alcohol consumption, physical activity, household income, CCI, fasting blood sugar, systolic blood pressure, total cholesterol, and antidepressant use were taken into consideration in the matching. A matching ratio of 1:1 was used, with a caliper of 0.1 times the standard deviation of the logit propensity score. After propensity score matching, standardized differences were calculated to assess the distribution of covariates between the antibiotic non-user group and the antibiotic user group.

Data collection, data mining, and statistical analyses were conducted using SAS 9.4 (SAS Institute, Cary, NC, United States), STATA ver. 13.0 (STATA Corporation, College Station, TX, United States), and R version 4.2.0 (R Foundation for Statistical Computing, Vienna, Austria). Statistical significance was defined as a *p*-value <0.05 in a two-sided manner. P for trend was calculated by the cumulative days of antibiotic exposure and the number of antibiotic classes, as a continuous variable, separately. P for interaction in Cox proportional hazards models was calculated by including interaction terms between antibiotic exposure and covariates.

## Results

The baseline characteristics of participants are shown in Table 1. A total of 313,161 individuals were analyzed, with a mean age of 54.9 and a male distribution of 57.3%. The number of incident cases was 5,386 for overall dementia, 4,126 for AD, and 694 for VD. Compared to antibiotic non-users, antibiotic users tended to be older, smoke less, drink less alcohol, have lower income, and have higher CCI values. Among antibiotic users, long-term users tended to be older, smoke less, drink less alcohol, have higher CCI values, and have more antidepressant use.

**TABLE 1:** Descriptive characteristics of the cohort study population.

	Total population	Antibiotic non-user	Antibiotic user		
			Number of cumulative days prescribed		
			1–30	31–90	≥ 91
Number of people	313,161	75,619	203,347	30,222	3,973
Dementia event, N					
Overall dementia	5,386	991	3,497	774	124
Alzheimer’s disease	4,126	744	2,700	588	94
Vascular dementia	694	143	424	110	17
Age, years, mean (SD)	54.9 (9.13)	54.0 (8.95)	54.9 (9.09)	56.6 (9.46)	57.5 (9.76)
Sex, N (%)					
Men	179,477 (57.3)	48,034 (63.5)	113,322 (55.7)	15,560 (51.5)	2,561 (64.5)
Women	133,684 (42.7)	27,585 (36.5)	90,025 (44.3)	14,662 (48.5)	1,412 (35.5)
Body mass index, kg/m^2^, N (%)					
<18.5	6,851 (2.2)	1,866 (2.5)	4,269 (2.1)	594 (2.0)	122 (3.1)
18.5 ≤ BMI < 23	112,758 (36.0)	28,605 (37.8)	72,528 (35.7)	10,270 (34.0)	1,355 (34.1)
23 ≤ BMI < 25	87,601 (28.0)	20,931 (27.7)	57,176 (28.1)	8,414 (27.8)	1,080 (27.2)
25 ≤ BMI	105,951 (33.8)	24,217 (32.0)	69,374 (34.1)	10,944 (36.2)	1,416 (35.6)
Smoking status, N (%)					
Never smoker	216,462 (69.1)	49,182 (65.0)	142,397 (70.0)	22,198 (73.4)	2,685 (67.6)
Past smoker	30,536 (9.8)	7,495 (9.9)	19,700 (9.7)	2,849 (9.4)	492 (12.4)
Current smoker	66,163 (21.1)	18,942 (25.1)	41,250 (20.3)	5,175 (17.1)	796 (20.0)
Alcohol consumption, times per week, N (%)					
None	179,705 (57.4)	40,194 (53.2)	117,735 (57.9)	19,236 (63.7)	2,540 (63.9)
≤2	100,865 (32.2)	26,586 (35.2)	64,712 (31.8)	8,473 (28.0)	1,094 (27.5)
≥3	32,591 (10.4)	8,839 (11.7)	20,900 (10.3)	2,513 (8.3)	339 (8.5)
Physical activity, times per week, N (%)					
None	155,776 (49.7)	37,489 (49.6)	101,022 (49.7)	15,237 (50.4)	2,028 (51.0)
1–4	123,485 (39.4)	30,656 (40.5)	80,054 (39.4)	11,274 (37.3)	1,501 (37.8)
5–7	33,900 (10.8)	7,474 (9.9)	22,271 (11.0)	3,711 (12.3)	444 (11.2)
Household income, N (%)					
First quartile (lowest)	44,881 (14.3)	11,142 (14.7)	28,763 (14.1)	4,401 (14.6)	575 (14.5)
Second quartile	65,655 (21.0)	15,781 (20.9)	42,760 (21.0)	6,270 (20.8)	844 (21.2)
Third quartile	88,269 (28.2)	20,588 (27.2)	57,844 (28.5)	8,698 (28.8)	1,139 (28.7)
Fourth quartile (highest)	114,356 (36.5)	28,108 (37.2)	73,980 (36.4)	10,853 (35.9)	1,415 (35.6)
Charlson comorbidity index, N (%)					
0	98,370 (31.4)	35,745 (47.3)	57,948 (28.5)	4,178 (13.8)	499 (12.6)
1	86,362 (27.6)	19,492 (25.8)	58,523 (28.8)	7,468 (24.7)	879 (22.1)
2 or more	128,429 (41.0)	20,382 (27.0)	86,876 (42.7)	18,576 (61.5)	2,595 (65.3)
Fasting blood sugar, mg/dL, mean (SD)	97.8 (28.28)	97.6 (27.79)	97.8 (28.21)	98.4 (29.98)	98.7 (27.43)
Systolic blood pressure, mmHg, mean (SD)	126.2 (17.02)	126.9 (17.33)	126.0 (16.93)	126.1 (16.9)	126.7 (16.82)
Total cholesterol, mg/dL, mean (SD)	198.4 (36.89)	198.0 (36.83)	198.4 (36.80)	199.4 (37.51)	197.5 (37.61)
Antidepressant use, N (%)					
No	296,361 (94.6)	73,424 (97.1)	192,223 (94.5)	27,254 (90.2)	3,460 (87.1)
Yes	16,800 (5.4)	2,195 (2.9)	11,124 (5.5)	2,968 (9.8)	513 (12.9)
Respiratory diseases, N (%)					
No	311,605 (99.5)	75,619 (100)	202,418 (99.5)	29,714 (98.3)	3,854 (97.0)
Yes	1,556 (0.5)		929 (0.5)	508 (1.7)	119 (3.0)
Urinary tract infections (UTI), N (%)					
No	311,096 (99.3)	75,619 (100)	202,106 (99.4)	29,521 (97.7)	3,850 (96.9)
Yes	2,065 (0.7)		1,241 (0.6)	701 (2.3)	123 (3.1)
Skin, soft tissue, bone, and joint infections (SSTBJ), N (%)					
No	311,559 (99.5)	75,619 (100)	202,231 (99.5)	29,807 (98.6)	3,902 (98.2)
Yes	1,602 (0.5)		1,116 (0.6)	415 (1.4)	71 (1.8)
Intra-abdominal infections (IAI), N (%)					
No	313,046 (100)	75,619 (100)	203,266 (100)	30,191 (99.9)	3,970 (99.9)
Yes	115 (0)		81 (0)	31 (0.1)	3 (0.1)
Other infectious diseases, N (%)					
No	331,744 (99.6)	75,619 (100)	202,601 (99.6)	29,691 (98.2)	3,833 (96.5)
Yes	1,417 (0.5)		746 (0.4)	531 (1.8)	140 (3.5)

Acronyms; SD, standard deviation; N, number of people.

**FIGURE 2:**
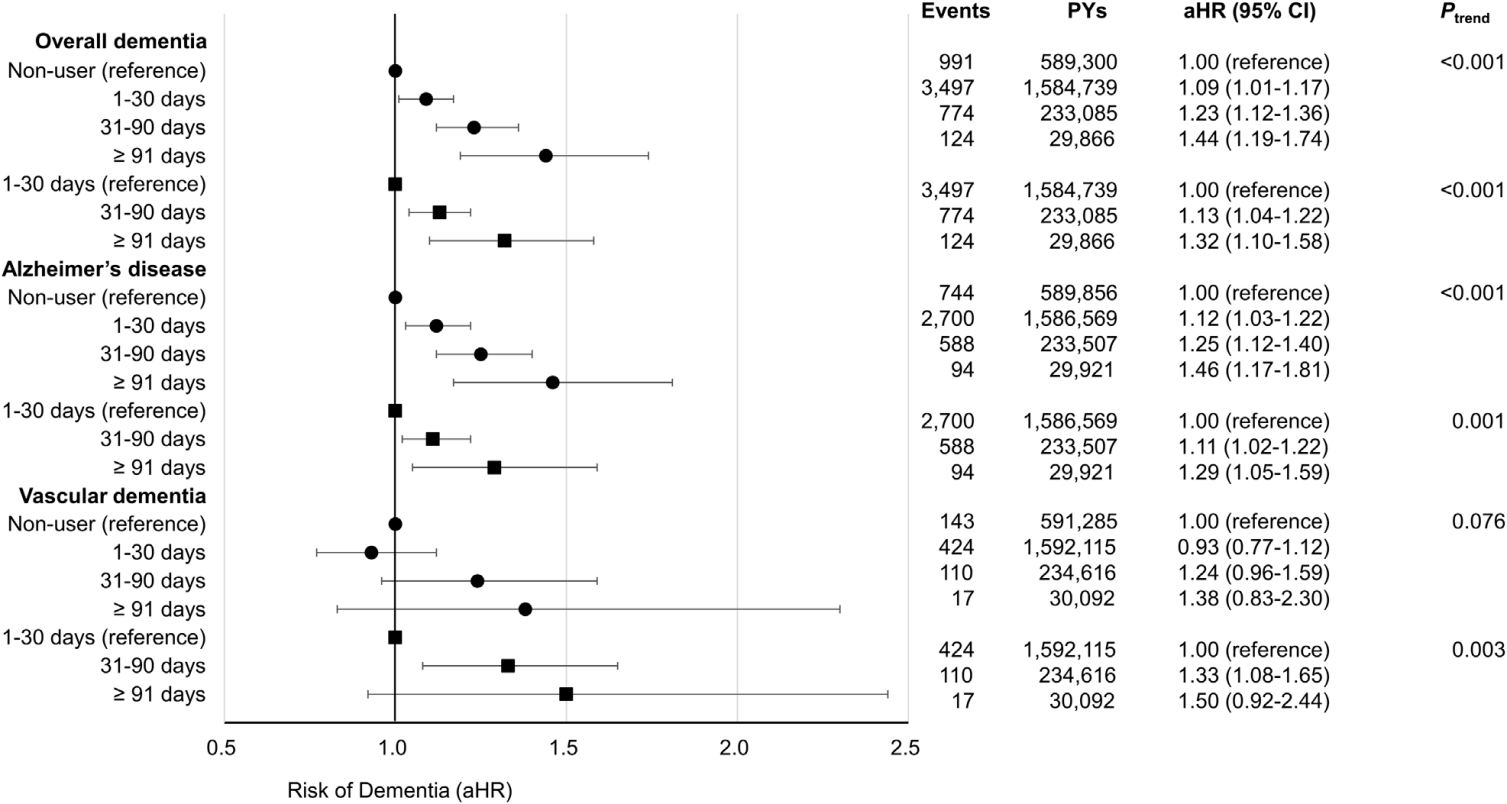
Forest plot indicates the association of cumulative days of antibiotic prescription with the risk of dementia. Acronyms; PYs, person-years; aHR, adjusted hazard ratio; CI, confidence interval. The aHRs were calculated by Cox proportional hazards regression after adjustments for age, sex, body mass index, smoking status, alcohol consumption, physical activity, household income, Charlson comorbidity index, fasting blood sugar, systolic blood pressure, total cholesterol, and antidepressant use. In these analyses, antibiotic non-users were considered as the reference group. The aHRs were calculated by Cox proportional hazards regression after adjustments for age, sex, body mass index, smoking status, alcohol consumption, physical activity, household income, Charlson comorbidity index, fasting blood sugar, systolic blood pressure, total cholesterol, antidepressant use, and infectious diseases (respiratory diseases, urinary tract infections, skin, soft tissue, bone and joint infections, intra-abdominal infections, and other infectious diseases). In these analyses, 1–30 cumulative days of antibiotics prescribed users were considered as the reference group.

The association between cumulative antibiotic prescription days and the risk of developing overall dementia, AD, and VD is depicted in Figure 2 and Supplementary Table S1. Compared to antibiotic non-users, antibiotic users, including users of 1–30, 31–90, 91 or more cumulative prescription days, had an increased risk of overall dementia (aHR, 1.09; 95% CI, 1.01–1.17 for 1–30 days, aHR, 1.23; 95% CI, 1.12–1.36 for 31–90 days, and aHR, 1.44; 95% CI, 1.19–1.74 for 91 or more days), AD (aHR, 1.12; 95% CI, 1.03–1.22 for 1–30 days, aHR, 1.25; 95% CI, 1.12–1.40 for 31–90 days, and aHR, 1.46; 95% CI, 1.17–1.81 for 91, or more days), and VD (aHR, 0.93; 95% CI, 0.77–1.12 for 1–30 days, aHR, 1.24; 95% CI, 0.96–1.59 for 31–90 days, and aHR, 1.38; 95% CI, 0.83–2.30 for 91 or more days) in a duration-responsive manner. There was a significant trend of risk increase for overall dementia and AD upon increasing levels of cumulative days of antibiotic prescription from non-users to users. The second model, additionally adjusted for infectious diseases, also showed a clear duration-responsive relationship between cumulative antibiotic prescription days with overall dementia (aHR, 1.13; 95% CI, 1.04–1.22 for 31–90 days and aHR, 1.32; 95% CI, 1.10–1.58 for 91 or more days), AD (aHR, 1.11; 95% CI, 1.02–1.22 for 31–90 days and aHR, 1.29; 95% CI, 1.05–1.59 for 91 or more days), and VD (aHR, 1.33; 95% CI, 1.08–1.65 for 31–90 days and aHR, 1.50; 95% CI, 0.92–2.44 for 91 or more days). The aHR for VD in antibiotic users for 91 or more days was not significant, but the trend for the increase of VD risk with increasing cumulative prescription days appeared significant (p for trend = 0.003). Therefore, a significant trend of risk increase was observed for overall dementia, AD, and VD upon increasing levels of cumulative prescription from short-term to long-term users.

Table 2 reports the stratified analysis according to subgroups of covariates for the association of antibiotic use with dementia. There was no interaction term by age, sex, BMI, smoking status, alcohol consumption, physical activity, household income, CCI, and antidepressant use.

**TABLE 2:** Stratified analyses of the association between antibiotic exposure and overall dementia incidence.

	Total	Event	aHR (95% CI) by the number of cumulative days antibiotics prescribed				*p* for interaction
			Non-user	1–30	31–90	≥ 91	
Age							0.054
< 60 years	220,912	551	1.00 (ref.)	1.17 (0.94–1.46)	1.47 (1.08–2.00)	1.79 (0.94–1.46)	
≥ 60 years	92,249	4,835	1.00 (ref.)	1.04 (0.96–1.12)	1.17 (1.05–1.29)	1.36 (1.12–1.66)	
Sex							0.130
Men	179,477	2,232	1.00 (ref.)	1.03 (0.93–1.15)	1.13 (0.98–1.32)	1.27 (0.97–1.67)	
Women	133,684	3,154	1.00 (ref.)	1.13 (1.03–1.25)	1.31 (1.16–1.49)	1.61 (1.24–2.10)	
Body mass index							0.873
< 25 kg/m^2^	207,210	3,653	1.00 (ref.)	1.08 (0.99–1.17)	1.28 (1.14–1.44)	1.32 (1.04–1.68)	
≥ 25 kg/m^2^	105,951	1,733	1.00 (ref.)	1.10 (0.96–1.25)	1.13 (0.95–1.35)	1.66 (1.21–2.26)	
Smoking status							0.124
Never	216,462	4,324	1.00 (ref.)	1.11 (1.02–1.20)	1.28 (1.15–1.42)	1.53 (1.24–1.89)	
Ever	96,699	1,062	1.00 (ref.)	1.02 (0.87–1.19)	1.08 (0.87–1.34)	1.17 (0.78–1.77)	
Alcohol consumption days/week							0.138
Never	100,865	832	1.00 (ref.)	0.99 (0.83–1.18)	0.97 (0.75–1.25)	1.36 (0.84–2.20)	
1 day or over	212,296	4,554	1.00 (ref.)	1.11 (1.02–1.20)	1.28 (1.15–1.42)	1.44 (1.18–1.77)	
Physical activity, times/week							0.706
Never	155,776	3,523	1.00 (ref.)	1.07 (0.98–1.16)	1.22 (1.08–1.37)	1.53 (1.22–1.92)	
1 time or over	157,385	1,863	1.00 (ref.)	1.12 (0.99–1.28)	1.25 (1.06–1.47)	1.24 (0.89–1.74)	
Household income							0.661
Lower half	110,536	2,345	1.00 (ref.)	1.11 (1.00–1.24)	1.23 (1.06–1.42)	1.47 (1.10–1.96)	
Upper half	202,625	3,041	1.00 (ref.)	1.06 (0.96–1.17)	1.23 (1.08–1.40)	1.40 (1.09–1.80)	
Charlson comorbidity index							0.502
0 or 1	184,732	1,838	1.00 (ref.)	1.11 (1.00–1.24)	1.20 (0.99–1.45)	1.47 (0.95–2.28)	
2 or over	128,429	3,548	1.00 (ref.)	1.10 (0.99–1.21)	1.26 (1.12–1.42)	1.45 (1.18–1.80)	
Antidepressant use							0.756
No	314,610	5,006	1.00 (ref.)	1.18 (1.10–1.28)	1.40 (1.26–1.55)	1.67 (1.35–2.06)	
Yes	17,867	774	1.00 (ref.)	0.96 (0.77–1.20)	1.20 (0.93–1.55)	1.31 (0.86–2.00)	

Acronyms; aHR, adjusted hazard ratio; CI, confidence interval; ref., reference. The aHRs were calculated by Cox proportional hazards regression after adjustments for age, sex, body mass index, smoking status, alcohol consumption, physical activity, household income, Charlson comorbidity index, fasting blood sugar, systolic blood pressure, total cholesterol, and antidepressant use.

In Table 3, we also conducted analyses by excluding 1-, 2-, and 3-year latent periods. The aHR for overall dementia in 91 or more days of antibiotic use was 1.53 (95% CI, 1.26–1.87) compared to the non-user group in the 3-year wash-out period analysis. Moreover, the aHR for AD in 91 or more days of antibiotic use was 1.56 (95% CI, 1.24–1.96). After shifting the index date from 1 January 2006 to 1 January 2007, those with antibiotic users for 91 or more days had a higher risk of overall dementia (aHR, 1.29; 95% CI, 1.12–1.49) and AD (aHR, 1.34; 95% CI, 1.13–1.58) compared to antibiotic non-users. Those with antibiotic users for 91 or more days had a higher risk of overall dementia (aHR, 1.29; 95% CI, 1.14–1.45) and AD (aHR, 1.27; 95% CI, 1.10–1.45) compared to those with antibiotic users for 1–30 days.

**TABLE 3:** Sensitivity analyses of the association between antibiotic exposure and dementia incidence.

	Total	Event	aHR (95% CI) by the number of cumulative days antibiotics prescribed			
			Non-user	1–30	31–90	≥ 91
Wash-out period after the index date						
Overall dementia						
No wash-out (Main)	313,161	5,386	1.00 (ref.)	1.09 (1.01–1.17)	1.23 (1.12–1.36)	1.44 (1.19–1.74)
1-year wash-out	312,993	5,218	1.00 (ref.)	1.10 (1.02–1.18)	1.25 (1.13–1.38)	1.45 (1.20–1.76)
2-year wash-out	312,759	4,984	1.00 (ref.)	1.10 (1.02–1.18)	1.25 (1.13–1.38)	1.49 (1.23–1.81)
3-year wash-out	312,408	4,633	1.00 (ref.)	1.10 (1.01–1.18)	1.28 (1.16–1.42)	1.53 (1.26–1.87)
Alzheimer’s disease						
No wash-out (Main)	313,161	4,126	1.00 (ref.)	1.12 (1.03–1.22)	1.25 (1.12–1.40)	1.46 (1.17–1.81)
1-year wash-out	312,993	3,981	1.00 (ref.)	1.12 (1.03–1.22)	1.26 (1.13–1.41)	1.47 (1.18–1.83)
2-year wash-out	312,759	3,786	1.00 (ref.)	1.12 (1.03–1.22)	1.28 (1.14–1.43)	1.53 (1.22–1.91)
3-year wash-out	312,408	3,511	1.00 (ref.)	1.12 (1.02–1.22)	1.30 (1.15–1.47)	1.56 (1.24–1.96)
Vascular dementia						
No wash-out (Main)	313,161	694	1.00 (ref.)	0.93 (0.77–1.12)	1.24 (0.96–1.59)	1.38 (0.83–2.30)
1-year wash-out	312,993	676	1.00 (ref.)	0.95 (0.78–1.16)	1.25 (0.96–1.62)	1.46 (0.88–2.43)
2-year wash-out	312,759	656	1.00 (ref.)	0.94 (0.77–1.15)	1.23 (0.94–1.60)	1.40 (0.83–2.36)
3-year wash-out	312,408	608	1.00 (ref.)	0.99 (0.80–1.22)	1.26 (0.95–1.66)	1.58 (0.94–2.68)
Variation of the exposure period and follow-up period						
5 years for exposure and 7 years for a follow-up						
Overall dementia						
Antibiotic non-user group as reference	320,128	5,442	1.00 (ref.)	1.00 (0.91–1.10)	1.23 (1.11–1.37)	1.29 (1.12–1.49)
1–30 days user group as reference	279,713	4,943		1.00 (ref.)	1.23 (1.16–1.31)	1.29 (1.14–1.45)
Alzheimer’s disease						
Antibiotic non-user group as reference	320,128	4,174	1.00 (ref.)	1.05 (0.94–1.18)	1.30 (1.15–1.46)	1.34 (1.13–1.58)
1–30 days user group as reference	279,713	3,810		1.00 (ref.)	1.23 (1.14–1.32)	1.27 (1.10–1.45)
Vascular dementia						
Antibiotic non-user group as reference	320,128	692	1.00 (ref.)	0.77 (0.61–0.98)	0.87 (0.66–1.14)	1.06 (0.73–1.56)
1–30 days user group as reference	279,713	610		1.00 (ref.)	1.11 (0.93–1.34)	1.36 (0.98–1.29)

Acronyms; aHR, adjusted hazard ratio; CI, confidence interval; ref., reference. The aHRs were calculated by Cox proportional hazards regression after adjustments for age, sex, body mass index, smoking status, alcohol consumption, physical activity, household income, Charlson comorbidity index, fasting blood sugar, systolic blood pressure, total cholesterol, and antidepressant use. The antibiotic non-user group was considered as a reference group. The aHRs were calculated by Cox proportional hazards regression after adjustments for age, sex, body mass index, smoking status, alcohol consumption, physical activity, household income, Charlson comorbidity index, fasting blood sugar, systolic blood pressure, total cholesterol, antidepressant use, respiratory diseases, urinary tract infections, skin, soft tissue, bone and joint infections, intra-abdominal infections, and other infectious diseases. The antibiotic 1–30 days user group was considered as a reference group.

The effect of the number of antibiotic classes prescribed on dementia incidence is demonstrated in Table 4. Using the non-user group as a reference, we revealed a positive association between the number of antibiotic classes and dementia including overall dementia and AD (p for trend <0.001). Furthermore, Supplementary Table S2 shows dementia risk to cephalosporin exposure. Both overall dementia (aHR, 1.14; 95% CI, 1.02–1.28) and AD (aHR, 1.17; 95% CI, 1.03–1.33) risks were notably higher in cephalosporin users compared to non-users. Supplementary Table S3 and Supplementary Table S4 show examples of each antibiotic class and the sources of infection based on ICD-10 codes.

**TABLE 4:** Risk for dementia incidence according to the number of antibiotic classes.

	Number of antibiotic classes prescribed						
	Antibiotic non-user	1	2	3	4	5 or more	*p* for trend
N of people (%)	75,619 (24.2)	95,297 (30.4)	79,788 (25.5)	44,706 (14.3)	15,337 (4.9)	2,414 (0.8)	
Overall dementia							
Events, N	991	1,545	1,477	948	361	64	
Person-years	589,300	742,427	620,835	346,972	118,713	18,742	
aHR (95% CI)	1.00 (ref.)	1.08 (1.00–1.17)	1.11 (1.03–1.21)	1.16 (1.06–1.27)	1.18 (1.05–1.34)	1.28 (1.00–1.66)	<0.001
Alzheimer’s disease							
Events, N	744	1,189	1,147	716	280	50	
Person-years	589,856	743,248	621,560	347,509	118,919	18,761	
aHR (95% CI)	1.00 (ref.)	1.11 (1.01–1.21)	1.15 (1.05–1.27)	1.17 (1.05–1.30)	1.22 (1.06–1.40)	1.34 (1.00–1.78)	<0.001
Vascular dementia							
Events, N	143	199	173	123	51	5	
Person-years	591,285	745,591	623,972	348,972	119,426	18,862	
aHR (95% CI)	1.00 (ref.)	0.97 (0.78–1.21)	0.91 (0.73–1.14)	1.05 (0.82–1.35)	1.18 (0.86–1.64)	0.72 (0.29–1.75)	0.573

Acronyms; N, number; aHR, adjusted hazard ratio; CI, confidence interval; ref., reference. The aHRs, were calculated by Cox proportional hazards regression after adjustments for age, sex, body mass index, smoking status, alcohol consumption, physical activity, household income, Charlson comorbidity index, fasting blood sugar, total cholesterol, and antidepressant use. Antibiotics were divided into seven classes consisting of penicillin, cephalosporin, macrolide, fluoroquinolone, sulfonamides, tetracyclines, and lincosamides or others.

Supplementary Tables S5, S6 depict the descriptive characteristics of the study population and the risk for dementia according to antibiotic exposure after propensity score matching, respectively. Antibiotic users had a higher risk of overall dementia and AD than non-users.

## Discussion

In this nationally representative cohort study, antibiotic use was associated with the risk of dementia incidence. There was a cumulative duration-dependent relationship between dementia incidences with cumulative days of antibiotic exposure. The results were significant after adjustments for all covariates. Furthermore, the results of the propensity score matching analysis verified our primary findings. To our knowledge, this is the first study to indicate that longer cumulative days of antibiotic exposure could lead to significantly higher dementia risk.

Previous studies showed that antibiotics can induce alterations in the gut microbiota, leading to AD (Angelucci et al., 2019; Liu et al., 2019). In a Chinese cohort, it has been demonstrated that the diversity of intestinal microbiota was significantly lower in AD patients (Liu et al., 2019). Enterobacteriaceae was enriched in AD patients compared with amnestic mild cognitive impairment patients and healthy control groups (Liu et al., 2019). In our study, antibiotic use was significantly correlated with dementia, and this could have been caused by the change in gut microbiota. Another previous study demonstrated that unhealthy lifestyle aspects can affect the gut microbiome (Pushpanathan et al., 2019). It has been suggested that a sedentary lifestyle can increase pro-inflammatory bacteria and the levels of Aβ in the brain, gradually causing a higher risk of AD (Askarova et al., 2020). Therefore, as lifestyle behavior could be a confounding factor, we included representative factors of lifestyle, such as smoking status (Anstey et al., 2007; Chen et al., 2009), alcohol consumption (Chen et al., 2009), and physical activity (Chen et al., 2009) as covariates. Our results of the stratified analyses on subgroups of these factors were consistent in all three subgroups, showing that lifestyle behaviors did not influence the effect of antibiotics on dementia.

Dementia is a chronic neurodegenerative disease with clinical characteristics such as slow onset and gradual progression (Arvanitakis et al., 2019; Dementia, 2019). In particular, dementia risk is linked to numerous diseases such as infections and head injury (van der Flier and Scheltens, 2005; Chen et al., 2009). Therefore, there is a possibility that antibiotics were prescribed due to the prognostic symptoms before the diagnosis of dementia. For instance, during the clinical course of dementia, the prevalence of dysphagia is high (Easterling and Robbins, 2008; Suh et al., 2009). It is likely to predispose individuals to aspiration pneumonia by dysphagia (Easterling and Robbins, 2008) and this may cause antibiotic prescription. In this case, antibiotics would have been prescribed for dementia, and it is difficult to assert that antibiotics induced dementia. Therefore, we considered CCI as a confounding factor and there was no significant interaction in a stratified analysis. Furthermore, we additionally adjusted for infectious diseases for analyses in the antibiotic user group considering the possibility of an association between infection and dementia incidence (Dunn et al., 2005). We used only one main principal diagnosis for infectious diseases to conduct accurate analyses. Thus, our results show that antibiotic prescription is an independent risk factor for future dementia risk.

Multiple mechanisms of the association between antibiotics and dementia risk have been postulated. First, the gut microbiota–brain axis is considered a crucial way that mediates the relationship between gut dysbiosis by antibiotics and dementia. A recent study has suggested that antibiotics can cause neuroinflammation and neurodegeneration through the gut microbiota–brain axis, favoring AD or worsening its course (Angelucci et al., 2019). Another study suggested the role of microbiota in the physiological and biological basis of neurodegenerative disorders in the gut–brain axis (Dinan and Cryan, 2017). Antibiotic exposure leads to short- and/or long-term changes in the gut microbiota such as reduced colonization resistance against pathogens, loss of diversity (Lange et al., 2016; Angelucci et al., 2019), and changes in brain chemistry (Bienenstock et al., 2015; Angelucci et al., 2019). The accumulation of antibiotics fosters bacterial resistance, and the human microbiome has become a reservoir of resistance genes (Francino, 2016). This causes prolonged dysbiosis of gut microbiota, explaining the increased risk of dementia due to long-term antibiotic use (Francino, 2016). Therefore, prolonged antibiotic use may cause a profound change in the gut–brain axis profoundly. Microbial exudates and amyloid-enhancing factors caused by impaired gut permeability and microbial dysbiosis enter the circulatory system, and this induces an abnormal level of pro-inflammatory cytokines (Doifode et al., 2020). These signals can cross the BBB, and peripheral immune cells activate glial cells and the neuroinflammatory pathways (Doifode et al., 2020). This process triggers tau accumulation and amyloid-beta burden that causes neurodegeneration (Doifode et al., 2020). Amyloid in the brain can activate microglia and astrocytes, resulting in inflammation (Doifode et al., 2020). Second, it has been demonstrated that overall antibiotics can cause neurotoxicity (Bhattacharyya et al., 2014), leading to encephalopathy (Bhattacharyya et al., 2016). As encephalopathy can present various symptoms including memory loss and dementia, this finding can partly explain the link between antibiotics and dementia. In particular, it is known that β-lactams such as penicillins and cephalosporins impede inhibitory neurotransmission at the ligand-gated ion channel γ-aminobutyric acid class A receptor (GABA_A_R), causing central excitotoxicity (Bhattacharyya et al., 2016). In our study, only cephalosporins among antibiotic classes were associated with a higher risk of dementia. In this regard, it is known that cephalosporins can induce nephrotoxicity in larger doses (Yılmaz and Özcengiz, 2017). It is becoming explicit that kidney diseases are affected by changes in the gut microbiota which produces excessive amounts of uremic toxins such as indoxyl sulfate but fewer renoprotective metabolites (Mahmoodpoor et al., 2017; Yang et al., 2018). Furthermore, in a cohort study with 3,207 participants, chronic kidney disease was significantly associated with dementia (Sasaki et al., 2011). Thus, gut microbiota dysbiosis by antibiotic exposure directly or indirectly may affect the incidence of dementia.

Our study had several limitations. First, the study population was confined to participants who underwent a national health examination. There is a possibility of selection bias that participants in a bad health have been excluded. Second, the operational definition of dementia was based on the claim data. Therefore, it has a validation problem about the real diagnoses although we defined the outcome by diagnosis and prescription concurrently. Thus, future studies with medical chart reviews are required. Another limitation concerns the follow-up duration of 8 years, which may not be long enough to fully disclose the effects of antibiotics on the risk of dementia. As dementia usually develops slowly with gradual cognitive decline (Arvanitakis et al., 2019; Dementia, 2019), future studies with a longer follow-up period are required to fully ascertain the conclusions of this research. Fourth, antibiotic exposure was defined by prescription. There is a possibility that some participants did not take antibiotics on the actual prescription. Moreover, information about lifestyle behaviors was collected from a self-reported survey. Thus, there is a possibility that the survey data had insufficient and unclear information. Furthermore, we could not consider the education level, which may be a primary risk factor (Chen et al., 2009), due to the lack of information in the database. Instead, we adjusted for the income level, which may be a surrogate marker of education. Furthermore, information about the exact changes in gut microbiota by antibiotics could not be investigated. In future studies, it is necessary to analyze its changes at both clinical and experimental levels considering factors that can change the gut microbiota. Finally, this study was retrospective cohort research. Therefore, this study does not prove causation but rather indicates an association between antibiotic exposure and dementia risk, even though we attempted to control for possible confounders and biases.

Despite these limitations, our study’s strengths include the ability to assess the association of antibiotic exposure with dementia risk in older adults. First, the NHIS-HEALS database was based on the medical claim records collected at the nationally representative level. It contains individual-level information about demographic data, health examinations, diagnoses, and prescriptions. Furthermore, a large study population of 313,161 may strengthen the generalization of our results. Second, we conducted the association between antibiotic exposure and dementia incidence by regarding various risk factors such as age, household income, CCI, and infectious diseases. Third, we conducted sensitivity analysis and propensity score matching analysis to determine the robustness of our findings. Analyses that considered latent periods to address reverse causality also showed consistent results.

Antibiotic exposure appears to be associated with greater dementia incidence in our cohort study. There was a cumulative duration-dependent association between antibiotic exposure and dementia risk. However, it is possible that individuals who were long-term prescribed antibiotics had a poor health status, which increases their vulnerability to dementia. In other words, antibiotic exposure might be a proxy for dementia risk factors that we could not consider in this study, not a direct cause of dementia. This study suggests that antibiotic exposure could be one of several risk factors for dementia. Ultimately, future prospective and epidemiological studies with a broader study population that investigate the association between changes in gut microbiota by antibiotic exposure and dementia incidence are needed to validate the interpretation of our findings.

## Data Availability

The data from the NHIS in South Korea are available to qualified researchers approved by the Korean National Health Insurance Service. Further information is available on the website (https://nhiss.nhis.or.kr).
