# Supplementary material for: Association between antibiotics and dementia risk: A retrospective cohort study
Source: Front Pharmacol. 2022 Sep 26;13:888333. doi: 10.3389/fphar.2022.888333 (PMC9548656; doi:10.3389/fphar.2022.888333)
Supplement: Supplementary file 1 [file DataSheet1.docx]

**Supplementary Material**

**Association between Antibiotics and Dementia Risk: A Retrospective Cohort Study**

**Supplementary Table S1. Risk for dementia by the number of cumulative days antibiotics prescribed.**

**Supplementary Table S2. Dementia risk by a single specific class of antibiotics class exposure compared to antibiotics non-user.**

**Supplementary Table S3. Examples of antibiotics for each class based on the World Health Organization Anatomical Therapeutic Chemical (WHO ATC) guidelines.**

**Supplementary Table S4. Representative sources of infection according to the ICD-10 codes, mainly categorized by systems.**

**Supplementary Table S5. Descriptive characteristics of the study population among antibiotics non-user group and antibiotics user group after propensity score matching.**

**Supplementary Table S6. Risk for dementia according to antibiotic exposure after propensity score matching.**

This supplementary material has been provided by the authors to give readers additional information about their work.

**Supplementary Table S1. Risk for dementia by the number of cumulative days antibiotics prescribed.**

|  |  | **Number of cumulative days antibiotics prescribed** | | |  | |
| --- | --- | --- | --- | --- | --- | --- |
|  | **Antibiotics non-user** | **1-30** | **31-90** | **≥ 91** | | ***p* for trend** |
| **Overall dementia** |  |  |  |  | |  |
| Events, N | 991 | 3,497 | 774 | 124 | |  |
| Person-years | 589,300 | 1,584,739 | 233,085 | 29,866 | |  |
| aHR (95% CI)^a^ | 1.00 (ref.) | 1.09 (1.01-1.17) | 1.23 (1.12-1.36) | 1.44 (1.19-1.74) | | < 0.001 |
| aHR (95% CI)^b^ |  | 1.00 (ref.) | 1.13 (1.04-1.22) | 1.32 (1.10-1.58) | | < 0.001 |
| **Alzheimer’s disease** |  |  |  |  | |  |
| Events, N | 744 | 2,700 | 588 | 94 | |  |
| Person-years | 589,856 | 1,586,569 | 233,507 | 29,921 | |  |
| aHR (95% CI)^a^ | 1.00 (ref.) | 1.12 (1.03-1.22) | 1.25 (1.12-1.40) | 1.46 (1.17-1.81) | | < 0.001 |
| aHR (95% CI)^b^ |  | 1.00 (ref.) | 1.11 (1.02-1.22) | 1.29 (1.05-1.59) | | 0.001 |
| **Vascular dementia** |  |  |  |  | |  |
| Events, N | 143 | 424 | 110 | 17 | |  |
| Person-years | 591,285 | 1,592,115 | 234,616 | 30,092 | |  |
| aHR (95% CI)^a^ | 1.00 (ref.) | 0.93 (0.77-1.12) | 1.24 (0.96-1.59) | 1.38 (0.83-2.30) | | 0.076 |
| aHR (95% CI)^b^ |  | 1.00 (ref.) | 1.33 (1.08-1.65) | 1.50 (0.92-2.44) | | 0.003 |

Acronyms; N, number of people; aHR, adjusted hazard ratio; CI, confidence interval; ref., reference.

^a^Reference group: antibiotics non-users

The aHRs were calculated by Cox proportional hazards regression after adjustments for age, sex, body mass index, smoking status, alcohol consumption, physical activity, household income, Charlson comorbidity index, fasting blood sugar, systolic blood pressure, total cholesterol, and antidepressant use.

^b^Reference group: 1-30 cumulative days of antibiotics prescribed user

The aHRs were calculated by Cox proportional hazards regression after adjustments for age, sex, body mass index, smoking status, alcohol consumption, physical activity, household income, Charlson comorbidity index, fasting blood sugar, systolic blood pressure, total cholesterol, antidepressant use, and infectious diseases (respiratory diseases, urinary tract infections, skin, soft tissue, bone and joint infections, intra-abdominal infections, and other infectious diseases).

**Supplementary Table S2. Dementia risk by a single specific class of antibiotics class exposure compared to antibiotics non-user.**

| **Exposure variable** | **Overall dementia** | | | **Alzheimer’s disease** | | | **Vascular dementia** | | |
| --- | --- | --- | --- | --- | --- | --- | --- | --- | --- |
|  | Total | Event | aHR (95% CI) | Total | Event | aHR (95% CI) | Total | Event | aHR (95% CI) |
| **Cephalosporin** |  |  |  |  |  |  |  |  |  |
| Antibiotics non-user | 75,619 | 991 | 1.00 (ref.) | 75,619 | 744 | 1.00 (ref.) | 75,619 | 143 | 1.00 (ref.) |
| User | 27,729 | 452 | **1.14 (1.02-1.28)** | 27,729 | 348 | **1.17 (1.03-1.33)** | 27,729 | 68 | 1.19 (0.89-1.60) |
| **Penicillin (including ampicillin and amoxicillin)** |  |  |  |  |  |  |  |  |  |
| Antibiotics non-user | 75,619 | 991 | 1.00 (ref.) | 75,619 | 744 | 1.00 (ref.) | 75,619 | 143 | 1.00 (ref.) |
| User | 36,860 | 527 | 1.07 (0.96-1.19) | 36,860 | 408 | 1.10 (0.97-1.24) | 36,860 | 60 | 0.84 (0.62-1.14) |
| **Fluoroquinolones** |  |  |  |  |  |  |  |  |  |
| Antibiotics non-user | 75,619 | 991 | 1.00 (ref.) | 75,619 | 744 | 1.00 (ref.) | 75,619 | 143 | 1.00 (ref.) |
| User | 17,052 | 358 | 1.07 (0.94-1.21) | 17,052 | 270 | 1.06 (0.92-1.22) | 17,052 | 44 | 0.97 (0.68-1.36) |
| **Macrolides** |  |  |  |  |  |  |  |  |  |
| Antibiotics non-user | 75,619 | 991 | 1.00 (ref.) | 75,619 | 744 | 1.00 (ref.) | 75,619 | 143 | 1.00 (ref.) |
| User | 10,569 | 173 | 1.06 (0.90-1.25) | 10,569 | 138 | 1.12 (0.93-1.35) | 10,569 | 22 | 0.96 (0.61-1.50) |
| **Tetracycline** |  |  |  |  |  |  |  |  |  |
| Antibiotics non-user | 75,619 | 991 | 1.00 (ref.) | 75,619 | 744 | 1.00 (ref.) | 75,619 | 143 | 1.00 (ref.) |
| User | 2,023 | 15 | 0.63 (0.38-1.05) | 2,023 | 10 | 0.56 (0.30-1.05) | 2,023 | 1 | 0.29 (0.04-2.04) |
| **Lincosamides** |  |  |  |  |  |  |  |  |  |
| Antibiotics non-user | 75,619 | 991 | 1.00 (ref.) | 75,619 | 744 | 1.00 (ref.) | 75,619 | 143 | 1.00 (ref.) |
| User | 277 | 4 | 1.10 (0.41-2.93) | 277 | 3 | 1.09 (0.35-3.40) | 277 | 1 | 1.98 (0.28-14.21) |
| **Sulfonamides** |  |  |  |  |  |  |  |  |  |
| Antibiotics non-user | 75,619 | 991 | 1.00 (ref.) | 75,619 | 744 | 1.00 (ref.) | 75,619 | 143 | 1.00 (ref.) |
| User | 787 | 16 | 1.38 (0.84-2.26) | 787 | 12 | 1.37 (0.77-2.43) | 787 | 3 | 1.81 (0.58-5.69) |

Acronyms; aHR, adjusted hazard ratio; CI, confidence interval; ref., reference.

The aHRs were calculated by Cox proportional hazards regression after adjustments for age, sex, body mass index, smoking status, alcohol consumption, physical activity, household income, Charlson comorbidity index, fasting blood sugar, total cholesterol, and antidepressant use.

**Supplementary Table S3. Examples of antibiotics for each class based on the World Health Organization Anatomical Therapeutic Chemical (WHO ATC) guidelines.**

| **Antibiotics class** | **Types of antibiotics for each class** |
| --- | --- |
| Macrolides | erythromycin, spiramycin, midecamycin, oleandomycin, roxithromycin, josamycin, troleandomycin, clarithromycin, azithromycin, miocamycin, rokitamycin, dirithromycin, flurithromycin, telithromycin, solithromycin |
| Penicillins | ampicillin, pivamicillin, carbenicillin, amoxicillin, amoxicillin and clavulanate, azlocillin, mezlocillin, mecillinam, piperacillin, ticarcillin, metampicillin, talampicillin, dicloxacillin, oxacillin |
| Cephalosporins | cefalexin, cefaloridine, cefalotin, cefazolin, cefatrizine, ceftezole, cefoxitin, cefotetan, cefonicid, cefotaxime, ceftazidime, ceftriaxone, cefmenoxime, cefdinir, cefteram, cefepime, cefpirome, cefaclor, cefuroxime |
| Fluoroquinolones | ofloxacin, ciprofloxacin, pefloxacin, enoxacin, temafloxacin, norfloxacin, levofloxacin, moxifloxacin, gemifloxacin, gatifloxacin, sitafloxacin |
| Sulfonamides | sulfaisodimidine, sulfamethizole, sulfadimidine, sulfapyridine, sulfafurazole, sulfanilamide, sulfathiazole, sulfathiourea, sulfamethoxazole and trimethoprim, sulfadiazine, sulfasalazine, sulfamoxole |
| Lincosamides | clindamycin, lincomycin |
| Tetracyclines | doxycycline, tetracycline |

**Supplementary Table S4. Representative sources of infection according to the ICD-10 codes, mainly categorized by systems.**

| **Infectious diseases** | **ICD-10 codes** |
| --- | --- |
| **Respiratory diseases** |  |
| Pneumonia & Influenza | J09, J10, J11, J12, J13, J14, J15, J16, J17, J18 |
| Chronic bronchitis | J41, J42 |
| **Urinary tract infections (UTI)** |  |
| Cystitis | N30 |
| Acute pyelonephritis | N10 |
| Urethritis | N34, N37 |
| **Skin, soft tissue, bone and joint infections (SSTBJ)** |  |
| Cellulitis | L03 |
| Erysipelas | A46 |
| Impetigo | L01 |
| Folliculitis | L66.2, L66.4 |
| Furuncle & Carbuncle | L02 |
| Osteomyelitis | M86 |
| Synovitis | M65, M67, M68, M70 |
| **Intra-abdominal infections (IAI)** |  |
| Cholecystitis & Cholangitis | K80, K81, K83 |
| Appendicitis | K35, K36, K37 |
| Diverticulitis | K57 |
| Peritonitis | K65 |
| Pancreatitis | K85 |
| **Others** |  |
| Acute/chronic otitis media | H65, H66 |
| Sepsis | A40, A41 |
| Central nervous system infection | A81, A89 |

**Supplementary Table S5. Descriptive characteristics of the study population among antibiotics non-user group and antibiotics user group after propensity score matching.**

|  | **Antibiotics**  **non-user** | **Antibiotics**  **user** | **Standardized difference** |
| --- | --- | --- | --- |
| **Number of people** | 75,598 | 75,598 |  |
| **Dementia Event, N** |  |  |  |
| Overall dementia | 991 | 1,172 |  |
| Alzheimer’s disease | 744 | 897 |  |
| Vascular dementia | 143 | 152 |  |
| **Antibiotic subscription days, mean (SD)** | 0 (0) | 16.32 (24.53) |  |
| **Age, years, mean (SD)** | 54.00 (8.95) | 54.51 (8.98) | 0.057 |
| **Sex, N (%)** |  |  |  |
| Men | 48,014 (63.51) | 43,494 (57.53) | 0.123 |
| Women | 27,584 (36.49) | 32,104 (42.47) |  |
| **Charlson Comorbidity Index, N (%)** |  |  |  |
| 0 | 35,724 (47.26) | 33,921 (44.87) | 0.171 |
| 1 | 19,492 (25.78) | 15,761 (20.85) |  |
| 2 or more | 20,382 (26.96) | 25,916 (34.28) |  |
| **Body Mass Index, kg/m^2^, N (%)** |  |  |  |
| < 18.5 | 1,864 (2.47) | 1,555 (2.06) | 0.1 |
| 18.5 ≤ BMI < 23 | 28,593 (37.82) | 25,654 (33.93) |  |
| 23 ≤ BMI < 25 | 20,928 (27.68) | 21,076 (27.88) |  |
| 25 ≤ BMI | 24,213 (32.03) | 27,313 (36.13) |  |
| **Smoking status, N (%)** |  |  |  |
| Never smoker | 49,175 (65.05) | 52,043 (68.84) | 0.083 |
| Past smoker | 7,494 (9.91) | 7,018 (9.28) |  |
| Current smoker | 18,929 (25.04) | 16,537 (21.87) |  |
| **Alcohol consumption, times per week, N (%)** |  |  |  |
| None | 40,190 (53.16) | 42,844 (56.67) | 0.072 |
| ≤ 2 | 26,578 (35.16) | 24,918 (32.96) |  |
| ≥ 3 | 8,830 (11.68) | 7,836 (10.37) |  |
| **Physical activity, times per week, N (%)** |  |  |  |
| None | 37,474 (49.57) | 35,772 (47.32) | 0.063 |
| 1-4 | 30,651 (40.54) | 31,041 (41.06) |  |
| 5-7 | 7,473 (9.89) | 8,785 (11.62) |  |
| **Household income, N (%)** |  |  |  |
| First quartile (lowest) | 11,130 (14.72) | 9,734 (12.88) | 0.081 |
| Second quartile | 15,776 (20.87) | 14,837 (19.63) |  |
| Third quartile | 20,584 (27.23) | 22,892 (30.28) |  |
| Fourth quartile (highest) | 28,108 (37.18) | 28,135 (37.22) |  |
| **Fasting Blood Sugar, mean (SD)** | 97.55 (27.29) | 97.01 (28.91) | 0.019 |
| **Systolic blood pressure, mmHg, mean (SD)** | 126.90 (17.32) | 125.31 (17.11) | 0.093 |
| **Total cholesterol, mean (SD)** | 198.02 (36.83) | 198.36 (36.55) | 0.009 |
| **Antidepressant use, N (%)** |  |  |  |
| No | 73,403 (97.10) | 71,891 (95.10) | 0.103 |
| Yes | 2,195 (2.90) | 3,707 (4.90) |  |

Acronyms; SD, standard deviation; N, number of people.

All standardized differences are absolute values.

**Supplementary Table S6. Risk for dementia according to antibiotic exposure after propensity score matching.**

|  | **Events** | **Person-years** | **aHR (95% CI)** |
| --- | --- | --- | --- |
| **Overall dementia** |  |  |  |
| Antibiotics non-user (n=75,598) | 991 | 589,163 | 1.00 (ref.) |
| Antibiotics user (n=75,598) | 1,172 | 589,819 | **1.09 (1.004-1.19)** |
| **Alzheimer’s disease** |  |  |  |
| Antibiotics non-user (n=75,598) | 744 | 589,718 | 1.00 (ref.) |
| Antibiotics user (n=75,598) | 897 | 590,446 | **1.11 (1.01-1.23)** |
| **Vascular dementia** |  |  |  |
| Antibiotics non-user (n=75,598) | 143 | 591,148 | 1.00 (ref.) |
| Antibiotics user (n=75,598) | 152 | 592,239 | 0.99 (0.79-1.25) |

Acronyms; N, number of people; aHR, adjusted hazard ratio; CI, confidence interval; ref., reference.

Propensity score matching was performed according to antibiotic exposure. Age, sex, body mass index, smoking status, alcohol consumption, physical activity, household income, Charlson comorbidity index, fasting blood sugar, systolic blood pressure, total cholesterol, and antidepressant use were taken into consideration upon matching. Using a caliper of 0.1 times the standard deviation of the logit propensity score, a matching ratio of 1:1 was used.

The aHRs were calculated by Cox proportional hazards regression after adjustments for age, sex, body mass index, smoking status, alcohol consumption, physical activity, household income, Charlson comorbidity index, fasting blood sugar, systolic blood pressure, total cholesterol, and antidepressant use.
